# Dry Eye Disease among Patients with Glaucoma under Topical Antiglaucoma Agents in a Tertiary Care Centre: A Descriptive Cross-sectional Study

**DOI:** 10.31729/jnma.7674

**Published:** 2022-09-30

**Authors:** Anjan Palikhey, Shahdev Koiree, Roshan Kumar Mehta, Amit Kumar Shrivastava

**Affiliations:** 1 Department of Pharmacology, Universal College of Medical Sciences, Bhairahawa, Rupandehi, Nepal; 2 Department of Pharmacy, Universal College of Medical Sciences, Bhairahawa, Rupandehi, Nepal

**Keywords:** *antiglaucoma agents*, *dry eye disease*, *glaucoma*, *prevalence*

## Abstract

**Introduction:**

Dry eye disease is a disorder of the tears and ocular surface that results in various symptoms, such as dryness, grittiness, burning and itching sensation in the eye, excessive tearing, and fluctuating vision. The prevalence of dry eye disease symptoms is increased with long-term topical anti-glaucoma medicine treatment. The objective of this study was to find out the prevalence of dry eye disease in glaucoma patients under topical anti-glaucoma agents in a tertiary care centre.

**Methods:**

A descriptive cross-sectional study was conducted from 9 February 2022 to 5 June 2022 among glaucoma patients under topical anti-glaucoma agents in a tertiary care centre after receiving ethical approval from the Institutional Review Committee (Reference number: UCMS/IRC/028/22). Data were collected using a standard ocular surface disease index questionnaire. The dry eye disease was clinically diagnosed if the ocular surface disease index score was equal to or more than 13. Point estimate and 95% Confidence Interval were calculated.

**Results:**

Among 250 glaucoma patients, dry eye disease was found in 180 (72%) (66.43-77.57, 95% Confidence Interval). Mild, moderate, and severe symptoms were present in 50 (27.78%), 68 (37.78%), and 62 (34.44%) of the patients, respectively. The most frequently observed dry eye symptoms were itching and irritation seen in 56 (31.11%), followed by pain in the eye in 41 (22.77%) and redness in the eye in 32 (17.77%) patients.

**Conclusions:**

The prevalence of dry eye disease was higher than other clinical studies done in similar setting. It is extremely concerning and calls for increased focus on managing glaucoma and concurrent dry eye disease.

## INTRODUCTION

Dry eye disease (DED) is a multifactorial disease of the tears, characterized by an inability to produce enough tears to moisten the ocular surface and accompanied by an increase in the osmolarity of the tear film and inflammation of the ocular surface. The prevalence of DED increases with the age, number, and duration of topical glaucoma treatments.^1,2^

Glaucoma is Nepal's third cause of blindness after cataracts and corneal conditions.^3^ Topical drugs such as prostaglandin analogues (bimatoprost, travoprost), beta-blockers (timolol), carbonic anhydrase inhibitors (dorzolamide, brinzolamide), and adrenergic agonists (brimonidine) are the mainstay of treatment. Preservatives in anti-glaucoma medications,particularly benzalkonium chloride (BAK), or the active drug itself, can lead to chronic ocular surface inflammation.^4,5^ DED can reduce patient adherence to glaucoma therapy, leading to treatment failure and compromising the patient quality of life.^6-8^

The main objective of this study was to find out the prevalence of dry eye disease among patients with glaucoma under topical antiglaucoma agents in a tertiary care centre.

## METHODS

This was a descriptive cross-sectional study conducted from 9 February 2022 to 5 June 2022 among glaucoma patients in the outpatient glaucoma department at Lumbini Eye Institute and Research Center (LEIRC). The ethical approval was taken from the Institutional Review Committee of the Universal College of Medical Science and Teaching Hospital (Reference number: UCMS/IRC/028/22). The official written permission was also taken from LEIRC for data collection from the glaucoma patients. Informed consent in written and verbal form was taken from the eligible participants.

Glaucoma patients, who had been under one or more topical intraocular pressure-lowering medications for at least 3 months, revisiting the glaucoma unit for the follow-up, were included in the study. Patients with recent ocular infection, lid abnormality, presence of immune-compromising disease, and any ocular surface lesions were excluded. A convenience sampling method was used. The sample size was calculated by using the following formula:


$$\begin{array}{ll} \text{n} & ={\text{Z}}^{2}\times \frac{\text{p}\times \text{q}}{{\text{e}}^{\text{2}}}\\ & =1.96^{2}\times \frac{0.797\times 0.203}{0.05^{2}}\\ & =249 \end{array}$$

Where,

n = minimum required sample size
Z = 1.96 at 95% Confidence Interval (CI)
p = prevalence of dry eye in glaucoma patients, 79.70%^9^
q = 1-p
e = margin of error, 5%

The minimum calculated sample size was 249. However, 250 participants were included in our study.

Socio-demographic variables of the participants, topical antiglaucoma agents used, and dry eye symptoms like itching, grittiness, burning sensation, blurring of vision, and redness experienced by the participants were recorded in a structured questionnaire format.

The data regarding the prevalence of dry eye disease was gathered through a face-to-face interview using a standard Ocular Surface Disease Index (OSDI) questionnaire.^10^ The index demonstrates sensitivity and specificity in identifying patients with dry eye symptoms from normal people. The OSDI is a reliable tool for quickly assessing the symptoms and the severity of dry eye disease (normal, mild, moderate, and severe) and its impact on vision-related function in the past week of a patient's life. Before conducting the actual study, the questionnaire was pretested on 5% of the sample size and these findings were not included in the final analysis.

The questionnaire was also translated into Hindi and Nepali to comprehend the participants better. The 12-item OSDI questionnaire includes questions on ocular symptoms, vision-related function, and environmental triggers. Each question is graded from 0 to 4, where 0 indicates none of the time; 1, some of the time; 2, half of the time; 3, most of the time; and 4, all of the time.

A well-trained optometrist helped to complete the questionnaire. A formula was then used to determine the final OSDI score.^10^ The OSDI score was graded for each participant on a scale of 0 to 100. An OSDI score ≥13 indicated a clinically relevant presence of DED. Participants were further grouped into the following categories by OSDI score: mild symptoms (13-22), moderate symptoms (23-32), and severe symptoms (33-100).^10^ Data were entered and analysed in IBM SPSS Statistics 20.0. Point estimate and 95% CI were calculated.

## RESULTS

Among 250 glaucoma patients, dry eye disease was found in 180 (72%) (66.43-77.57, 95% CI). Mild symptoms were present in 50 (27.78%) (Table 1).

**Table 1:** Distribution of dry eye disease using OSDI score among glaucoma patients (n= 18)

OSDI score (Severity of symptoms)	n (%)
OSDI score 13-22 (Mild symptoms)	50 (27.78)
OSDI score 23-32 (Moderate symptoms)	68 (37.78)
OSDI score 33-100 (Severe symptoms)	62 (34.44)

Males were 104 (57.78%) and females were 76 (42.22%). The age group most afflicted by glaucoma was ≥60 years 78 (43.34%), followed by 40-59 years age group 69 (38.33%). The mean age of the participants was 54.39±13.81 years. A total of 159 (88.30%) participants used two or more topical medications for lowering intraocular pressure (Table 2).

**Table 2:** Characteristics of patients with dry eye disease (n= 180).

Variables	n (%)
**Age**	
18-39 years	33 (18.33)
40-59 years	69 (38.33)
≥ 60 years	78 (43.34)
**Gender**	
Male	104 (57.78)
Female	76 (42.22)
**Education status**	
Illiterate	81 (45.00)
Primary level	37 (20.60)
Secondary level	54 (30.00)
Undergraduate level	8 (4.40)
**Occupation**	
Housewife	56 (31.10)
Farmer	43 (23.90)
Employed	48 (26.70)
Unemployed	33 (18.30)
**Number of topical medications used**	
Single	21 (11.70)
Two or more	159 (88.30)

The most common prescribed anti-glaucoma agent was brimonidine+timolol maleate 126 (70.00%), followed by dorzolamide 92 (51.11%) and bimatoprost 75 (41.66%). The least prescribed medications were travoprost+timolol two (1.11%) and brinzolamide+brimonidine two (1.11%) (Table 3).

**Table 3:** Topical antiglaucoma agents prescribed for patients with dry eye disease (n= 180).

Drugs	Preservatives used (w/v)^*^	n (%)
Brimonidine+timolol maleate	0.01% BAK^†^	126 (70.00)
Dorzolamide	0.0075% BAK	92 (51.11)
Bimatoprost	0.02% BAK	75 (41.66)
Timolol maleate	0.01% BAK	23 (12.77)
Brimonidine tartrate	0.005% SOC^‡^	13 (7.22)
Brinzolamide + brimonidine	0.02% BAK	2 (1.11)
Travoprost+timolol	0.01% BAK	2 (1.11)

* w/v = weight/volume

† BAK= Benzalkonium Chloride

‡ 800= Stabilised Oxychloro Complex

The most common symptoms of dry eyes among patients were itching and irritation seen in 56 (31.11%), followed by pain in the eye seen in 41 (22.77%) (Table 4).

**Table 4:** Symptoms of dry eye disease (n=180).

Dry eye symptoms	n (%)
Itching and Irritation	56 (31.11)
Pain in eye	41 (22.77)
Redness in eye	32 (17.77)
Blurred and poor vision	16 (8.88)
Grittiness	6 (3.33)
Burning sensation	3 (1.66)

## DISCUSSION

Topical antiglaucoma agents are always the first line of treatment for glaucoma. However, prolonged use of these agents has the potential to alter the cornea and conjunctiva, leading to dry eye syndrome. Although the prevalence of dry eye has been estimated to be around 25% out of 1599 patients attending the Opthalmology outpatient department of Dhulikhel Hospital,^11^ the present study found that DED was prevalent in 72.00% of glaucoma patients. The ocular surface is adversely affected by chronic topical intra ocular pressure lowering treatment.

There are several additional reasons why DED was more prevalent in our study. The present study was conducted among glaucoma patients attended at the glaucoma unit of Lumbini Eye Institute and Research Center (LEIRC), Siddharthanagar. LEIRC, a referral tertiary eye hospital, receives most of its patients from other institutions owing to DED symptoms or signs. Another reason could be that most of our patients are elderly with a mean age of 54.39 years, and it is generally known that dry eye is more prevalent in older people. In the previous studies done in Ethiopia and Japan, the prevalence was similar and was 76.00% and 73.50%, respectively.^12,13^

Our study results were different from the previously conducted studies, where the prevalence of DED in glaucoma patients was 54.30%, 42.10% and 45.10%, respectively.^14-16^ The results of these studies could be different since DED was defined using different standards and measurement techniques. The disparity in prevalence may be explained by the fact that the Glaucoma Symptom Scale rather than the OSDI questionnaire was utilized in the Rossi investigation. Climate fluctuations, environmental factors like pollution, or different sample populations may affect the results. Patients in developed countries are also more likely to have access to medications without preservatives, which implies they will experience fewer dry eye symptoms.

Housewives made up about 31.10% of the sample size in this study. About 23.90% of the population are farmers who toil long hours in adverse circumstances. They endure oppressive heat, sunlight, wind, and dust. It is also impossible to overlook how much exposure to pesticides and fertilizers increases farmers' risk of developing DED.^17^

Brimonidine and timolol maleatefixed-dosecombination (FDC) was the most commonly prescribed topical antiglaucoma agent, followed by Dorzolamide.^18^ In the same line, in a study conducted in an Indian tertiary care hospital, it was reported that the most commonly prescribed FDCs were timolol and brimonidine, followed by dorzolamide.^18^ A total of 72.22% of participants in the current study had moderate to severe dry eye symptoms, which comprised the majority of the DED symptoms experienced by the participants. One of the most important predictors of DED severity is the number of lOP-lowering medications used.^19^ In the present study, 88.30% of the participants used two or more topical antiglaucoma agents to lower intraocular pressure.

According to several other studies conducted in different clinical settings, BAK, a preservative found in most medications, has been linked to DED symptoms in patients. BAK has adverse effects on the conjunctiva and cornea like subclinical inflammation, dysfunction of the corneal epithelial barrier, tear film instability and an increased prevalence of dry eye symptoms.^5,20,21^ In the present study, most glaucoma patients received more than two BAK-containing medications, which led to a higher level of overall preservative concentration that may be contributing to DED symptoms. Few studies have demonstrated that DEDs are brought on by active drugs rather than the excipients used as preservatives. Because of this, most research suggested that using medications without preservatives could help reduce the symptoms of DED.^22^

The common symptoms of DED among most patients in the present study were itching, irritation, redness, pain in the eyes, and blurred vision, whereas grittiness and burning sensation were reported in a few patients. In support of our study, another previous study reported similar findings of adverse response symptoms in patients receiving anti-glaucoma agents.^23^

This study had its limitations. The number of participants was relatively small and performed for a short duration. Second, only a questionnaire method was utilized to ascertain the prevalence of DED; additional criteria, such as tear break-up time and the Schirmer test, would have increased the significance of the findings.

## CONCLUSIONS

The prevalence of dry eye disease among glaucoma patients under topical antiglaucoma agents was higher when compared to other studies conducted in similar settings. An ophthalmologist should be aware of this and treat any dry eye symptoms observed in patients to ensure that they continue taking their glaucoma medication as prescribed.
